# Association of Frailty Status with Risk of Fall among Hospitalized Elderly Patients: A Cross-Sectional Study in an Acute Geriatric Unit

**DOI:** 10.3390/medicines9100048

**Published:** 2022-09-20

**Authors:** Abrar-Ahmad Zulfiqar, Perla Habchi, Ibrahima Amadou Dembele, Emmanuel Andres

**Affiliations:** 1 Service de Médecine Interne, Diabète et Maladies Métaboliques de la Clinique Médicale B, Hôpitaux Universitaires de Strasbourg, Université de Strasbourg, 67000 Strasbourg, France; 2 Anesthesiology Consultant, Aman Hospital, F Ring Rd, Zone 47, Building 412, Doha P.O. Box 8199, Qatar

**Keywords:** risk of fall, elderly, frailty, comprehensive gerontological assessment

## Abstract

Introduction: The objective was to study the association of frailty status in hospitalized elderly patients with risk of fall in an acute geriatric unit and to characterize elderly “fallers” using a comprehensive gerontological assessment. Patients and Methods: A cross-sectional study was conducted in patients over 65 years of age and hospitalized in an acute geriatric unit. This work was carried out in the Acute Geriatric Medicine Unit, Saint-Julien Hospital, Center Hospitalier Universitaire de Rouen from 1 June 2016 to 15 August 2016. Results: 172 patients were included during the collection period, with a female predominance of 115 patients (66.9%). The average age of the sample was 79.37 years old (65–85). The average CHARLSON score was 6.93 (3–16). Patients came from home in 81.4% of cases (i.e., 140 patients), and from a nursing home in 18.6% of cases (i.e., 32 patients). The risk of falling, as assessed by the Monopodal Support Test, returned as abnormal for 127 patients. In our series, there was a statistically strong link between the risk of falling and the presence of a dementia pathology (*p* = 0.009), the presence of a vitamin D deficiency (*p* = 0.03), the presence of frailty, as assessed by the three scales (modified SEGA scale, Fried scale and CFS/7 (<0.001), a high comorbidity score (*p* = 0.04), and a disturbed autonomy assessment according to IADL (*p* = 1.02 × 10^−5^) and according to ADL (*p* = 6.4 × 10^−8^). There was a statistically strong link between the risk of falling and the occurrence of death (*p* = 0.01). Conclusion: The consequences of the fall in terms of morbidity and mortality and the frequency of this event with advancing age and its impact on the quality of life as well as on health expenditure justify a systematic identification of the risk of falling in the elderly population. It is therefore important to have sensitive, specific, and reproducible tools available for identifying elderly people at high risk of falling.

## 1. Introduction

Falling is defined as involuntarily ending up on the ground or in a lower position than the starting position. Falls in the elderly are a very common medical occurrence. They are a geriatric disorder in their own right and multifactorial events requiring a global approach that takes the elderly patient’s state of health, behavior, and environment into account. Falling is a major public health problem in the elderly due to its frequency and associated individual and collective consequences. A fall often reveals a complex medical, social, and even ethical situation, with problems with the environment, orientation, or at-home living. Falls are the prerogative of the elderly subject, revealing or creating frailty [1]. The incidence and severity of complications secondary to a fall increase with age, and mortality and morbidity are greater in the elderly [1]. Between 30% and 40% of people over 65 fall each year [1]. According to a review of randomized controlled studies [2], 30–50% of people over 65 fall each year. Half of people over the age of 80 living at home fall at least once a year [3]. Of these falls, 20% lead to medical intervention, with 9–10% causing a fracture [2]. More recent studies confirm the occurrence of 30% of people over the age of 65 living at home falling at least once a year and 15% falling at least twice, which translates into approximately 500 falling incidents per 1000 patient-years [4]. The recidivism rate within a year is also high, with one in two relapsing. Incidence increases with age and is more common in women than in men [5].

After a fall, a third of elderly people develop a fear of falling [6], which is accompanied by disturbances in balance, walking, cognition, and reduced mobility [6] that can precipitate an additional fall. It can even develop into a psychomotor regression syndrome. Falls represent 5–10% of admissions to medical emergency departments and are the third leading cause of admission in acute geriatric medicine. According to the EPAC survey, conducted in 2004 in France, 450,000 people over the age of 65 went to the emergency room following a fall, and 37% of them were hospitalized for a short stay. An increase in the hospitalization rate with age has been noted, going from 27% between 65 and 69 years old to 44% over 90 years old [7]. The consequences of falls in terms of morbidity and mortality, the frequency of this event with advancing age, and its impact on quality of life as well as on healthcare expenditures justify the systematic identification of the risk of falls in the elderly population. It is therefore important to have sensitive, specific, and reproducible tools available for identifying elderly people at a high risk for falling. Because of the physical, psychological, psychomotor, and social consequences, the consumption of medical goods and services is significantly increased in fall patients. Indeed, falls can lead to consultations in the emergency room, medical and nursing care, surgical interventions, hospitalizations, physiotherapy care, the need for home help, and can sometimes even lead to the institutionalization of the falling patient. In this context, the overall assessment of the elderly subject from the first fall is of particular importance. It is in this spirit that recent recommendations regarding the falling of elderly persons [7] underscore the need to set up a true evaluation process for the risk of falling as well as its management and prevention. Prevention covers a set of actions “aimed at reducing the impact of the determinants of diseases or health problems, avoiding the occurrence of diseases or health problems, stopping their progression or limiting their consequences. Preventive measures may consist of medical intervention, environmental control, legislative, financial or behavioral measures, political pressure or health education” [8]. The identification and assessment of older people at risk of falling is important because it allows for the implementation of preventative programs adapted to each older person.

With this in mind, we conducted this work to study, among subjects hospitalized in an acute geriatric unit, the risk of falling under the prism of a standardized gerontological assessment and an assessment of fragility. The objective was to study the association of frailty status in hospitalized elderly patients at risk of falling in an acute geriatric unit and to characterize elderly fallers through a standardized gerontological assessment.

## 2. Method

### 2.1. Type of Study

This was a cross-sectional study of patients over the age of 65, hospitalized in an acute geriatrics unit. This work was carried out in the Acute Geriatric Medicine Unit, Saint-Julien Hospital, Center Hospitalier Universitaire de Rouen, from 1 June 2016 to 15 August 2016.

### 2.2. Inclusion-Exclusion Criteria

Our study population consisted of patients aged 65 or older who were hospitalized in the Acute Geriatric Medicine Unit, Saint-Julien Hospital during the period of data collection.

Patients labeled as receiving palliative care when entering the service were excluded.

### 2.3. Data Collection

For each patient hospitalized in the department during this period, we collected:

− Sex and age, reason for hospitalization, medical and surgical history, Charlson comorbidity score, and incidence of hospitalization over the past two years.
− On the social level, the origin of the place of life.
− Biometrics, consisting of height (the use of heel/knee height when standing measurements was not possible) and weight. From these values, we were able to calculate the Body Mass Index (BMI) from the formula, i.e., weight (in kg)/(height × height (in m). Regarding its interpretation, we used the normal HAS BMI values if greater than or equal to 24 kg/m^2^. For a multifactorial approach, we also used the MNA to define whether the patients were properly nourished (score greater than 24), at risk of malnutrition (score between 17 and 24) or malnourished (score below 17).
− Data from the Comprehensive Geriatric Assessment (CGA) were also collected, consisting of the single-leg support test, which was defined as normal if the value was greater than or equal to five seconds. Dependence was assessed using the Katz and Lawton scales (ADL and IADL). Memory disorders were assessed by the MMSE score. The study of thymia was carried out with the help of the mini GDS, with a score greater than or equal to 1 indicating the presence of a high probability of depression.
− From a biological point of view, we noted the values of the assessment of entry into the department carried out on D1 by the nurses: albumin (hypoalbuminemia was defined by an albuminemia strictly lower than 35 g/L), the clearance of creatinine (kidney failure was defined by creatinine clearance MDRD < 60 mL/min/1.73 m^2^), hemoglobin (anemia was defined by hemoglobin <12 g/dL), TSH (hypothyroidism was defined by a TSH >4 microU/mL and hyperthyroidism by a TSH < 0.4 microU/mL), and vitamin D levels (a vitamin D deficiency corresponds to a rate lower than 30 ng/mL).
− To define frailty, we used the Fried score [9], the modified SEGA (mSEGA) part A score [10], and the Rockwood “Clinical Frailty Scale” scale rated out of 7 [11]. With the Fried scale, non-frail people score a 0, pre-frail or intermediate score between 1 and 2, and frail people score a 3. Using the SEGA part A score, a score lower than or equal to 8 indicate a person who is not very frail, a score higher than 8 and less than or equal to 11 describes a frail person, and a score greater than 11 indicates a very frail person. We used the Clinical Frailty Scale (CFS) in our study, evolving from the Canadian Study of Health and Aging. It was developed as a grading tool with seven scales in 2005 [11]: 1—Very Fit; 2—Well; 3—Managing Well; 4—Living With Very Mild Frailty; 5—Living with Mild Frailty; 6—Living With Moderate Frailty; 7—Living With Severe Frailty. For scores of 5 or more, the elderly patient was considered by CFS to be “frail”.

### 2.4. Statistical Analysis

Data were analyzed using SAS software version 9.4 (SAS Institute Inc., Cary, NC). The qualitative variables were described in the form of counts and percentages, and the quantitative variables in the form of an average. We conducted a comparative analysis between subjects with and without the risk of fall using Fisher’s exact test and the Chi-2 test. The multivariate analysis was conducted with the Cox model with an ascending “step-by-step” selection method of the candidate variables. The significance level of the statistical tests carried out was set at *p* < 0.05 for all the tests.

### 2.5. Administrative Elements

From a regulatory standpoint, informed consent was obtained from all patients included in this study. From an ethical and regulatory point of view, the study obtained authorization from the National Commission for Computing and Liberties (Number 2094245 v 0) as well as from the Committee for the Protection of Persons (registration slip: ID number BCR: 2017-A02727-46).

## 3. Results

### 3.1. General Results

Data from 172 patients were gathered during the collection period, with a female predominance of 115 subjects (66.9%). The average age of the sample was 79.37 years old (65–85). The average CHARLSON score was 6.93 (3–16). Of the patients, 140 (81.4%) came from home, while 32 (18.6%) came from a nursing home. Medical histories were dominated by cardiovascular pathologies for 145 patients (84.3%). Medical and surgical history details are indicated in Table 1. The detail of the treatments by therapeutic class and by molecule in our series is indicated in Table 1 and Table 2. The reason for admission of the elderly subjects in the series was dominated by the deterioration of their general state in 44 cases (25.6%) and a fall in 43 cases (25%). Details of the main reasons for admission are shown in Table 1. In total, 90 patients in the series had been hospitalized during the two years preceding the current admission to the acute geriatric unit.

### 3.2. Results of the Biological Variables of the Series

In our sample, hypoalbuminemia was detected for 81 patients (47.1%), renal failure for 59 patients (34.3%), hypothyroidism for 13 patients (7.5%), anemia for 80 patients (46.5%), and, finally, vitamin D deficiency for 125 patients (72.7%).

### 3.3. Consequences of a Fall in the Sample

In our sample, 43 elderly patients were hospitalized following a fall. Biological rhabdomyolysis with elevated CPK levels was detected in 32 subjects (74.4%) without renal failure or hydroelectrolyte disorders. A fracture consequence was found for four patients (two fractures of the ischio-iliopubic branch, an elbow fracture and a shoulder fracture), for whom only orthopedic treatment was recommended. A post-fall syndrome was detected for 10 subjects (23.25%).

### 3.4. Aftermath of a Fall in the Sample

Of the patients, 94 returned to their place of residence, i.e., 54.6% of the sample (73 patients at home and 21 patients in their nursing home), while 57 patients (33.1%) were admitted for recovery. There were 15 deaths during hospitalization and 22 deaths after hospitalization, for a total of 39 deaths in this sample. Another hospitalization was necessary for 39 patients (22.67%).

### 3.5. Statistical Analysis

In our series, there is a statistically strong link between the risk of falling and the presence of dementia (*p* = 0.009), the presence of a vitamin D deficiency (*p* = 0.03), the presence of frailty as assessed by the three scales (<0.001), a high comorbidity score (*p* = 0.04), a disturbed autonomy assessment according to IADL (*p* = 1.02 × 10^−5^) and according to ADL (*p* = 6.4 × 10^−8^). There is a statistically strong link between the risk of falling and the occurrence of death (*p* = 0.01) (Table 3).

In our study, a patient with vitamin D deficiency is 3.30x more likely to have a risk of falling, a patient with impaired autonomy is 15.03x more likely to fall, and a frail patient according to the SEGA score is at a 1.17x risk of falling (Table 4) Age is also associated with the risk of falling (*p* = 0.001), but not the use of benzodiazepins (*p* = 0.132), neuroleptics (*p* = 0.456), and Parkinson therapies (*p* = 0.155) in our study.

## 4. Discussion

In our series, there was a statistically strong link between the risk of falling and the presence of frailty as assessed by the three scales (<0.001), whether it was the Fried scale, the modified SEGA score part A, or the CFS scale. Very little work has been done in studying the link between the risk of falling and the frailty syndrome in an acute geriatric unit. In South Korea, Kim YS et al. conducted a longitudinal study over a period of twelve years, between 2006 and 2018, using data from the Korean Longitudinal Study of Aging. Frailty was measured using the Korean Frailty Scale, and fall event data were collected during follow-up visits. Of elderly subjects up to 65 years of age, those with frailty had a higher risk of falling than those without [12].

Frailty was more strongly associated with a higher risk of future falls among community-dwelling older people in a Korean meta-analysis [13]. Frailty is usually accompanied by low bone mineral density, decreased muscle mass, and chronic inflammation, all of which are typical risk factors for falling [14]. Fall prevention should be a part of a frail person’s daily routine. Improved quality of life and reduced physical symptoms may contribute to preventing falls. It is true that during hospitalization in an acute geriatric unit, current practices focus on the reason for hospitalization, while geriatric issues tend to take a back seat. Early detection of geriatric risks and problems can improve functional outcomes in these patients [15]. Multidimensional interventions on physical, nutritional, psychological, and social domains are effective and can prevent negative health outcomes [16]. In the Netherlands, systematic screening for frailty is performed by nurses upon hospital admission [17,18] through the use of several frailty scales. The risk of falling increases with age and is estimated to be 1.16 to 3.6x higher in individuals already affected by frailty syndrome [19]. Reduced muscle strength and body mass, fear of falling and the resulting slowdown of gait speed appreciably contribute to the development of frailty syndrome as well as to sustaining falls [20]. In The Netherlands, data from the Longitudinal Aging Study Amsterdam which looked at 311 community-dwelling participants, aged 75 years and older who participated in the three-year longitudinal study, showed that the risk of falling was higher in frail compared with non-frail adults, but no effect modification was seen for frailty on the association between physical activity and falls [21]. An observational and longitudinal study of 781,081 individuals living in Wales was conducted between 1 January 2010 and 31 December 2020 and found similar results with frailty increasing the risk of falling [22].

The origin of frailty is multifactorial and considered a risk factor for the occurrence of adverse events and dependence in the elderly. The causes of frailty syndrome are not yet fully understood. The pathophysiological factors which affect the development of frailty syndrome embrace abnormal metabolic processes, disorders of the endocrine and immune systems, coagulation disorders, and the musculoskeletal system in addition to both obesity and malnutrition [23]. It is associated with an appreciable rise in the overall risk of falling, reduced self-reliance in the pursuit of routine activities of daily life (ADLs), frequent hospitalization, and death [24]. We found a link at the limit of significance between depression, according to the mini GDS, and the risk of falling. Studies point in this direction. Recently, in 2022, longitudinal data from three biennial waves of the Health and Retirement Study (HRS) between 2010 and 2014 revealed that major depression was associated with significantly greater odds of experiencing a fall, an injury from a fall and multiple falls over a two-year period. Frailty was a significant mediator of the effects of depression on singular and multiple falls [25].

### Limits of the Study

One of the limits of our work lies in the evaluation of the risk of falling in an acute situation during the hospitalization of our subjects. It would have been interesting to conduct an assessment of frailty at a certain time (three and/or six months) after hospitalization, according to the three scales mentioned above. The outcome of these patients three and six months after hospitalization would be also studied. This will be the subject of further research. Another limitation to note is the monocentric nature of our study.

## 5. Conclusions

The consequences of falls in terms of morbidity and mortality, the frequency of this event with advancing age, and its impact on the quality of life as well as on health expenditures justify a systematic identification of the risk of falling in the elderly population. It is therefore important to have sensitive, specific, and reproducible tools available for identifying elderly people at a high risk of falling. The production and evaluation of information on the risks of falls and the interest of screening and targeting patients and their entourages could allow for improvements with respect to primary and secondary prevention.

## Figures and Tables

**Table 1:** Description of the sample population.

		N = 172
Sex, n (%)	Female	115 (66.9)
	Male	57 (33.1)
Age, m		79.37 (65–85)
Charlson, out of 24, m (sd)		6.93 (3–16)
**Medical history, n (%)**		
Cardiac disease		145 (84.3%)
Cognitive disorder		50 (29%)
Diabetes		47 (27.3%)
Neurological disease		47 (27.3%)
Neoplasm		39 (22.6%)
Pulmonary disease		33 (19.2%)
Articular prothesis		32 (18.6%)
Hemopathy		7 (4%)
**Drugs**		
Number of treatments, n (%)		
Antihypertensives		134 (77.9%)
Antidepressants		72 (41.8%)
Antiplatelet agents		71 (41.3%)
Statins		69 (40.1%)
Benzodiazepins		68 (39.5%)
Pump proton inhibitors		66 (38.4%)
Painkillers		58 (33.7%)
Anticoagulants		32 (18.6%)
Lthyroxin		28 (16.3%)
Vitamin D		25 (14.5%)
Antiarythmics		23 (13.4%)
Insulin		21 (12.2%)
Oral diabetics		20 (11.6%)
Parkinson therapies		11 (6.4%)
**Nature of hospitalization, n (%)**		
Deterioration of the general state		44 (25.6%)
Fall		43 (25%)
Neurological etiology		30 (17.4%)
Pulmonary etiology		23 (13.4%)
Others		19 (11%)
Home stay difficult		16 (9%)
Hematological etiology		14 (8%)
Confusion		12 (7%)
Nephrological etiology		12 (7%)
Cardiological etiology		8 (4.6%)
**Geriatric criterion**		
Weight		65.87 kgs (32.7–108)
Height		1.58 m (1.34–1.82)
BMI		26.50 (11.73–55.14)
MNA		22.06 (6–29)
MMSE		17.17 (0–30)
ADL		3.53 (0–6)
IADL		2.83 (0–8)
MINIGDS		2.44 (0–4)
Monopodal test < 5 s		127 (73.8%)
**Biological metrics**		
Albumin		35.94 (22–64)
Creatinine		103.74 (27–583)
Glycemia		6.04 (0.69–14.3)
TSH		2.33 (0.01–60)
Hemoglobin		11.9 (7.6–17.1)
Vitamin D		21.94 (5–77)
**Frailty**		
Fried, out of 5, m		3.33 (0–5)
SEGA modified, out of 26, m		13.52 (2–24)
Rockwood, out of 7, m		5.44 (1–7)

BMI: Body Mass Index; MMSE: Mini Mental State Examination; MNA: Mini Nutritional Assessment; ADL: Activity of Daily Living; IADL: Instrumental Activity of daily living; MiniGDS: Mini Geriatric Depression Scale; TSH: thyroid-stimulating hormone; SEGA: Short Evaluation Geriatric Assessment.

**Table 2:** Therapeutics list.

Therapeutics List	Drugs
**Antihypertensives**	Diuretics: 66 patients (38.4%)
	Beta blockers: 59 patients (34.3%)
	ACE inhibitors: 48 patients (27.9%)
	Sartans: 38 patients (22.1%)
	Calcium channel blockers: 39 patients (22.7%)
	Central antihypertensives: 11 patients (6.4%)
**Antidepressants**	Serotoninergic reuptake inhibitors: 37 patients (21.5%)
	Other antidepressants: 36 patients (21%)
**Painkillers**	Level 1: 48 patients (27.9%)
	Level 2: 11 patients (6.4%)
	Level 3: 7 patients (4%)
**Neuroleptics**	Sedatives: 13 patients (7.5%)
	Anti productives: 10 patients (5.8%)
**Oral antidiabetics**	Metformin: 12 patients (7%)
	Sulfonamides: 7 patients (4%)
	Repaglinide: 8 patients (4.6%)
	New oral antidiabetics: 10 patients (5.8%)

**Table 3:** Comparison of certain variables of interest according to the risk of fall.

	Risk of Fall − n = 45	Risk of Fall + n = 127	*p*-Value
BMI (kg/m^2^)	26	26	0.33
Undernourishment	17 (37.8%)	59 (46.4%)	0.11
Cognitive disorders	25 (55.5%)	25 (19.7%)	1.51 × 10^−5^
Vitamin D deficiency	26 (57.7%)	99 (77.9%)	0.01
Mini GDS (/4)	2.1	2.1	0.08
mSEGA (/26)	9.8	14.8	<0.001
Fried (/5)	2.3	2.3	<0.001
CFS (/7)	4.6	5.8	<0.001
Charlson	6.8	6.8	0.04
Death	4	35	0.01
ADL	6	75	6.4 × 10^−8^
IADL	20	20	1.02 × 10^−5^
Benzodiazepins	16	52	0.6
Antidepressants	18	54	0.86
Hospitalization in the past two years	25	65	0.73
New hospitalization	11	28	0.84

BMI: Body Mass Index; Mini GDS: Mini Geriatric Depression Scale; CFS: Clinical Frailty Scale; ADL: Activity of Daily Living; IADL: Instrumental Activity of daily living.

**Table 4:** Multivariate model.

	OR	CI 95%	*p*-Value
Cognitive disorders	1.12	0.40–2.96	0.82
Vitamin D deficiency	3.30	1.24–9.08	0.017
ADL	15.03	5.46–41.32	<0.0001
mSEGA	1.17	1.03–1.33	0.012
Fried	1.27	0.92–1.77	0.14
CFS	1.38	0.92–2.08	0.11
Charlson	1.02	0.82–1.28	0.86

ADL: Activity Of Daily Living; mSEGA: modified Short Evaluation Geriatric Assessment; CFS: Clinical Frailty Scale. OR: Odds Ratio; CI: confidence interval.

## Data Availability

The datasets used and/or analyzed during the current study are available from the corresponding author upon reasonable request.
