# Correction: Zulfiqar et al. Association of Frailty Status with Risk of Fall among Hospitalized Elderly Patients: A Cross-Sectional Study in an Acute Geriatric Unit. *Medicines* 2022, *9*, 48

**DOI:** 10.3390/medicines13010005

**Published:** 2026-02-02

**Authors:** Abrar-Ahmad Zulfiqar, Perla Habchi, Ibrahima Amadou Dembele, Emmanuel Andres

**Affiliations:** 1 Service de Médecine Interne, Diabète et Maladies Métaboliques de la Clinique Médicale B, Hôpitaux Universitaires de Strasbourg, Université de Strasbourg, 67000 Strasbourg, France; 2 Anesthesiology Consultant, Aman Hospital, F Ring Rd, Zone 47, Building 412, Doha P.O. Box 8199, Qatar

## Text Correction

### Changes to Section 2.5. Administrative Elements

There was an error in the original publication [1]. The authors would like to clarify the ethical approval statement because of scientific clarity.

A correction has been made to Section 2.5, “Administrative Elements”. The corrected sentence is below:

From an ethical and regulatory point of view, the study obtained authorization from the CNIL (National Commission for Computing and Liberties, Number 2094245 v 0) on 28 August 2017, following an approval process that spanned several months due to administrative procedures.

### Changes to Institutional Review Board Statement

Following up on the clarification of ethical approval in the Methods Section, a change has been made to the Institutional Review Board statement. The correct sentence is given below:

This study was conducted according to the guidelines of the Declaration of Helsinki and received authorization from the CNIL (National Commission for Computing and Liberties, Number 2094245 v 0) on 28 August 2017.

The authors state that the scientific conclusions are unaffected. This correction was approved by the Academic Editor. The original publication has also been updated.
